# Supplementary material for: Human-robot collaborative task planning using anticipatory brain responses
Source: PLoS One. 2023 Jul 11;18(7):e0287958. doi: 10.1371/journal.pone.0287958 (PMC10335656; doi:10.1371/journal.pone.0287958)
Supplement: S1 Appendix — (PDF) [file pone.0287958.s001.pdf]

S1 Appendix - Validation of robot motion timing To determine the system latency or timing jitters between robot action onset and parallel port event trigger, a timing validation experiment was conducted. A photosensitive diode sensor was mounted at the center of the robot end-effector positioned approximately 0.5 cm above the monitor and facing the experiment screen. The experiment paradigm color scheme was altered to provide a deeper contrast for light measurement. A white circle with radius of 7 mm was added to each grid tile space (7 x 7 tiles) to provide an initial light reading for robot starting and end positions. The experiment scenario was then conducted as normal with the robot moving the LED mounted on the end-effector between grid tiles. The emitted light was recorded over an AUX channel of the EEG amplifier alongside the trigger indicating the onset of robot movements. This way, a latency distribution between robot movement onset and the parallel port trigger sent for EEG epoching could be determined over the course of one experiment.
