# Supplementary material for: Human-robot collaborative task planning using anticipatory brain responses
Source: PLoS One. 2023 Jul 11;18(7):e0287958. doi: 10.1371/journal.pone.0287958 (PMC10335656; doi:10.1371/journal.pone.0287958)
Supplement: S2 Appendix — (PDF) [file pone.0287958.s002.pdf]

S2 Appendix - Statistical testing To test for significant differences between conditions, non-parametric permutation testing was applied to all test-cases [74]. This method is particularly suitable for high-dimensional and highly correlated neural data as it does not a-priory assume a data distribution, but rather it derives the null-hypothesis distributions from the data itself. In addition, it does not require the individual tests to be independent, since the test results are derived from the entire correlated data itself (compare to the requirements/assumptions of the classical *t-test*). Furthermore, a variant of the method, suitable for multivariate data, proposes the derivation of global null-hypothesis distributions, e.g. among all variables, to account, i.e., correct for multiple comparisons. The method was applied applied according to the following steps: First, the true grand average contrast between the conditions which are tested against each other, e.g. between the non-error and the error condition, is computed. Second, we shuffle the labels of both conditions on the single-trial level for each subject. From this, a surrogate grand average contrast between the two conditions is computed. By randomly permuting the labels, this surrogate represents a contrast in which all previous information in the data is removed. From this multivariate surrogate (e.g. channels x time-points) the minimum value and the maximum value is determined and stored. Next, the derivation of surrogates is repeated for multiple times (in our case 50 times); each time the minimum and maximum value among all variables of the surrogate is stored. These values are subsequently used as the minimum and maximum null-hypothesis distribution. From the minimum distribution the 2.5% percentile and from the maximum distribution the 97.5% percentile is determined. These two values represent the lower and upper $p = 5\%$ threshold against which each individual variable of the true grand average is compared. If the value of a single variable falls below the 2.5% percentile of the minimum null-hypothesis distribution or falls above the 97.5% percentile of the maximum null-hypothesis distribution, this variable is considered significantly different at the $p = 5\%$ level between conditions.
