# Supplementary material for: Human-robot collaborative task planning using anticipatory brain responses
Source: PLoS One. 2023 Jul 11;18(7):e0287958. doi: 10.1371/journal.pone.0287958 (PMC10335656; doi:10.1371/journal.pone.0287958)
Supplement: S3 Appendix — (PDF) [file pone.0287958.s003.pdf]

S3 Appendix - Per subject results of ERP responses and single-trial classification performance

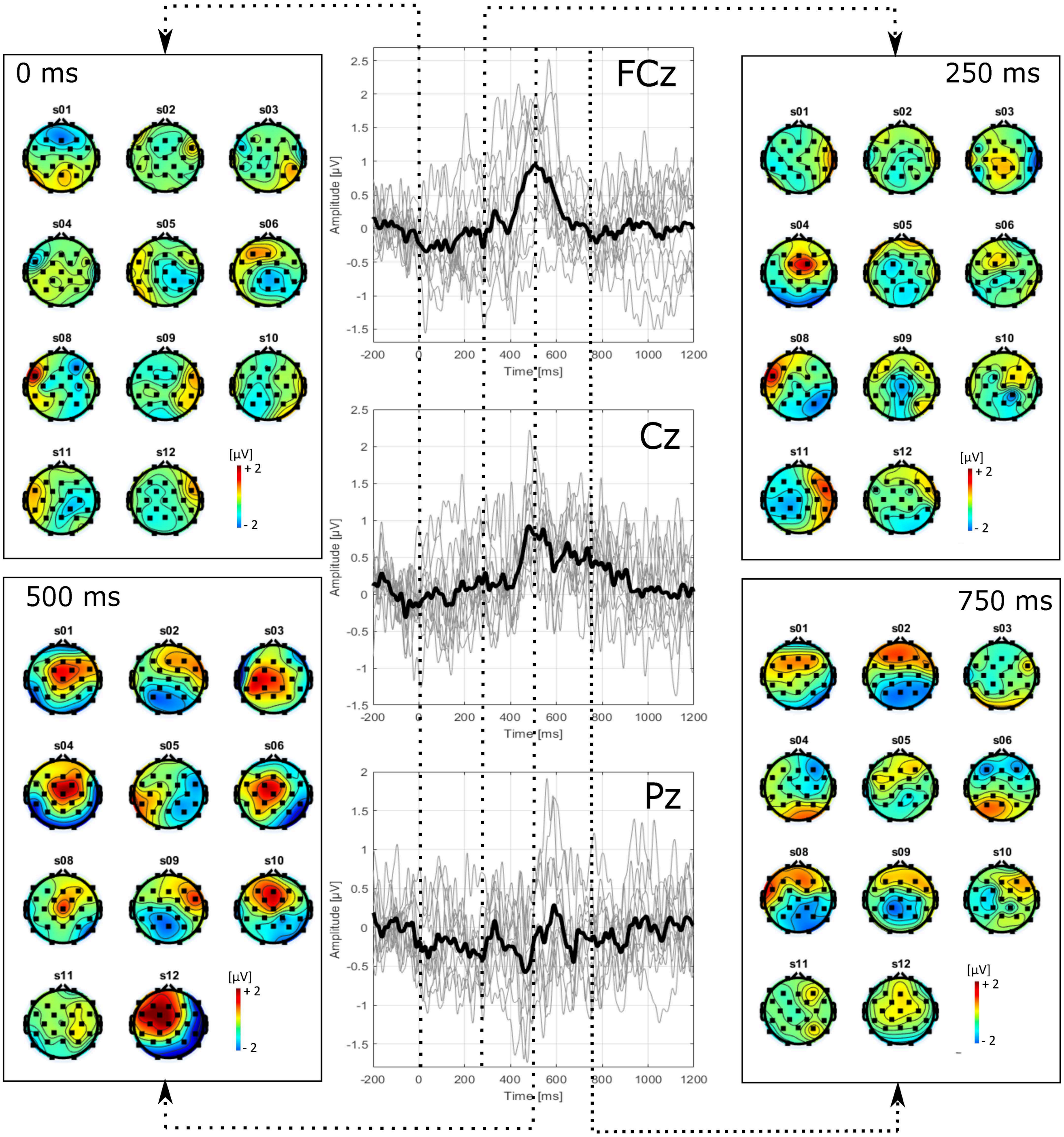

S3 Fig 1. Per subject ERP responses before anticipated human-to-robot takeover during intermittent collaboration: The middle panels show the per subject average (thin gray lines) and across subject grand average (thick black lines) difference ERP time courses of a frontal (FCz), central (Cz) and parietal (Pz) channel prior to a human takeover from robot (RH) versus robot continuation (RR) during intermittent collaboration. The side panels depict per subject topographic patterns at specific latencies time locked to the onset of robot end-effector movement. Time-domain and topographic patterns are most consistent across subjects for latencies around 500 ms.

S3 Table 1. Results of single-trial decoding performance during sequential collaboration: Per subject single-trial classification performance [%] and subject-specific sample-size adjusted chance-level ACC_{chance} for data of the *sequential collaboration* scenario for decoding anticipation of variants of takeover (HR, RH) against non-takeover situations (HH, RR). Results larger than the subject-specific chance-level are highlighted in bold.

ID	TNR_{HH}	TPR_{HR}	ACC_{HHvsHR}	ACC_{chance}	TNR_{RR}	TPR_{RH}	ACC_{RRvsRH}	ACC_{chance}
s01	**54.22**	**55.29**	**54.37**	54.07	52.00	50.10	51.73	54.46
s02	52.77	53.33	52.87	54.33	50.02	46.87	49.56	54.21
s03	**58.63**	**57.60**	**58.47**	54.12	**55.75**	**57.79**	**56.06**	53.99
s04	52.87	53.75	53.03	53.93	53.34	56.60	53.92	54.38
s05	51.91	54.79	52.31	53.67	51.26	52.03	51.40	54.78
s06	**54.61**	**56.88**	**54.91**	54.05	49.46	46.20	48.97	54.63
s08	54.97	47.31	53.66	54.18	**58.07**	**64.43**	**59.24**	54.54
s09	46.37	42.74	45.76	54.13	**54.87**	**54.97**	**54.89**	54.37
s10	48.04	45.73	47.73	54.26	53.13	50.69	52.68	54.40
s11	48.97	52.40	49.42	54.23	51.08	51.03	51.04	54.34
s12	48.69	46.03	48.24	54.57	52.85	56.02	53.38	54.24
MEAN	52.00	51.44	51.89	54.14	52.89	53.34	52.99	54.39
SD	3.65	5.08	3.72	0.23	2.57	5.30	2.99	0.21

S3 Table 2. Results of single-trial decoding performance during intermittent collaboration: Per subject single-trial classification performance [%] and subject-specific sample-size adjusted chance-level ACC_{chance} for data of the *intermittent collaboration* scenario for decoding anticipation of variants of takeover (HR, RH) against non-takeover situations (HH, RR). Results larger than the subject-specific chance-level are highlighted in bold.

ID	TNR_{HH}	TPR_{HR}	ACC_{HHvsHR}	ACC_{chance}	TNR_{RR}	TPR_{RH}	ACC_{RRvsRH}	ACC_{chance}
s01	53.81	55.43	54.12	54.30	**56.23**	**53.77**	**55.31**	54.24
s02	53.11	52.96	53.08	54.55	**59.61**	**58.83**	**59.30**	54.05
s03	49.93	49.91	49.93	54.07	**54.99**	**54.09**	**54.58**	54.37
s04	44.50	48.13	45.47	54.58	52.92	54.44	53.39	54.14
s05	52.78	52.77	52.79	54.22	**55.90**	**55.76**	**55.83**	53.99
s06	49.12	48.47	48.90	54.52	**64.32**	**62.92**	**63.72**	54.22
s08	50.57	48.35	49.94	54.37	**55.73**	**54.92**	**55.36**	54.02
s09	47.83	46.57	47.55	54.09	**55.62**	**54.87**	**55.38**	54.21
s10	**55.12**	**52.11**	**54.54**	54.35	54.62	53.32	54.08	54.13
s11	54.38	52.76	53.97	54.44	**59.53**	**53.29**	**57.05**	54.29
s12	**54.80**	**58.09**	**55.56**	54.31	**66.89**	**60.40**	**64.04**	54.52
MEAN	51.45	51.41	51.44	54.34	**57.85**	**56.06**	**57.09**	54.20
SD	3.37	3.49	3.26	0.17	4.34	3.21	3.70	0.16
