# Supplementary material for: Human-robot collaborative task planning using anticipatory brain responses
Source: PLoS One. 2023 Jul 11;18(7):e0287958. doi: 10.1371/journal.pone.0287958 (PMC10335656; doi:10.1371/journal.pone.0287958)
Supplement: Attachment — Submitted filename: Respose to Reviewers.pdf [file pone.0287958.s006.pdf]

Dear Editor and reviewers,

We thank the editor and reviewers for their constructive and insightful comments and recommendations which have helped further to significantly improve the manuscript. We have revised the manuscript (R2) according to all recommendations and carried out the corrections as suggested. Please find individual replies to the reviewer's comments. All changes to the manuscript are indicated in the responses and marked in blue in the 'Revised Manuscript with Track Changes'. Please note that line number references are valid only for the 'Revised Manuscript with Track Changes'. A separate clean version without tracked changes, named 'Manuscript', also accompanies this revision.

In addition, we decided to change the title of the manuscript to "Human-robot collaborative task planning using anticipatory brain responses", as we believe this reflects the work more adequately and will attract a broader audience.

Reviewer 3:

The authors investigate the characteristics of anticipatory movement-related brain activities in a human-robot interaction (HRI) scenario and how the neuro-cogitive measurements can improve robots' learning abilities. The manuscript is well written and organized. The authors have also addressed most of the comments from the previous reviewers. However, some of the details of experiment paradigm and definitions are still missing and thus reduce the value of the manuscript. I have some remarks for the authors.

1. I strongly recommend the authors to include a figure of experiment paradigm to better explain the experiment. When should the human/ robot initiate actions (key pressing)? Is there a signal telling human when one can initiate actions? Does robot always initiate actions at certain time point? If yes, is it right after the robot arm reaches the center of the grid or there is a delay? If no, what is the range of time period?

> *Response*: Thank you for the comment. We updated Fig. 1 to explain both scenarios, trial-by-trial in more detail. The figure illustrates in the *sequential Collaboration (sC)* scenario, that human and robot initiate the actions only in their area of responsibility, marked with different colors. In the intermittent Collaboration (iC), the human and robot handover is based on the direction of the next trajectory tile. When the robot initiates the actions, a new movement starts once the robot has arrived within a 2mm radius at the center of the tile. For more details, please see the answer to comment 4. These details are elaborated in Section 2.2 Experimental Paradigm in various locations (lines 186-235, changes are marked in blue in manuscript with tracked changes).

2. In Page 6, line 220. The authors mention the robot (or human) could mistakenly takeover in situations and robot mistakes occur at a rate of 30%. How does robot make mistakes if they are programmed to

initiate actions only when it is necessary? Does it mean that there is a process to control when the robot should initiate actions for the incoming trial? If yes, what is the criteria and when the robot will initiate actions (back to comment 1)?

> *Response*: The robot is programmed to initiate actions according to the answer to comment 1 (see above). In addition, the robot is programmed to randomly make mistakes (move in another direction than indicated by key press or “intended” by the robot) at a probability of 30%, independently if the next movement should have been initiated by the human or the robot. However, the brain responses to these mistakes are not the focus of the current study (please see the answer to the next question).

3. Following comment 2, Did the authors take out those mistake trials from both human and robot to prevent mixing anticipatory movement-related brain signals with error-related brain signals in the following analysis?

> *Response*: That is correct; only non-error trials were using for the analyses performed in this work to avoid mixing anticipatory brain signals with error-related brain signals. This information is now added more prominently in Section 2.5.1, page 9, line 347.

4. Related to comment 1. In page 7, line 264. The authors mention they randomized the robot arm movement duration to prevent subject habituate to robot arm movement onset. Does the robot arm move to next grid right after receiving actions from either robot or human, or there is a constant/ random delay between receiving actions and movement onset?

> *Response*: Thank you for addressing this point. We added additional clarity around robot movement duration (page 8, line 271). The robot movement duration was randomly set at the beginning of a session in the range of 0.6-1.2s. The movement duration is defined based on a start and end position at the center of grid spaces. However, additional random deviations from this movement duration arise from the robot completing a trial once within the 2mm radius of the end position (page 8, line 272), which depends on variations of preceding events. For example, a human may be slower to respond to the robot reaching the end position compared to the robot directly making movements. Once the robot is within the 2mm radius of the center of the grid, a trial is considered complete. At which point, a new trial begins with no delay (either robot takes action or human takes action after reacting). Therefore, the combination of preceding events and the 2mm radius make up the random robot movement duration per trial.

5. In page 8, line 293. The authors explain how artifact comtaminated EEG channels are removed by kurtosis and interpolated from neighboring channels. However, the definition of threshold 2% is unclear. Also, the interpolations method need to be further explained (linear, spline, etc.).

Response: Thank you for this comment. We clarified the statement by replacing the sentence with: “Next, we identified contaminated EEG channels using normalized kurtosis with a threshold of 2 (std. dev.) and subsequently used spherical interpolation to reconstruct rejected channels from the signals of neighboring electrodes.” (see page 8, line 302).

6. In page 9, line 344. The authors state the number of the trials in sC and iC scenario are approximately equal. Does it mean that the trajetory to the target is not the shortest path and thus it can pass the bolder where takeovers happen several times in sC scenario? I do not remember anything in the manuscript related to how the trajetory is defined.

Response: Thank you for the pointer. The trajectory to the target was not the shortest path, but it was randomly designed to contain at least one direction change (for example, first going to the right and afterwards going down) and at least 7 tiles. We expanded the parts in *2.2 Experimental paradigm* to make it clearer (see pages 5-6, lines 194, 204, 221).

7. In page 12, line 468. Should the sentence be "a robot takeover situation from human"?

Response: Thank you for the pointer. That is correct and has been changed accordingly (see page 12, line 481)

8. In page 13, line 504. The authors explain the simulation setup. However, there is no clear definition of which subtasks robot should take responsibility for (what is the correct subtasks for robot?). Are the correct subtasks defined as the subtasks not taken by human?

Response: The human choses a random subset of the n available subtasks unbeknownst to the robot, whereby the number of subtasks per partner are predefined in all simulations. The robot assigned subtasks are naturally the remaining subtasks which the human has not chosen. The robot’s task is to infer the assigned subtasks from the human EEG anticipatory responses. We changed the following sentence to the paragraph to clarify the explanations: “R's assigned subtasks are naturally the remaining subtasks which the human has not chosen.” (see page 14, line 518).

9. Related to comment 8. In page 18, line 710. The authors claim that imbalanced assignment of subtasks does not affect the learning. I am curious if the robot can learn faster (achieve higher Acc in early episode) when human chooses only 2 out of 10 comparing to 8 out of 10 since the number of "correct subtasks" for robot is less in the later condition. If there is no difference, the authors statement can be strengthen.

Response: Thank you for this comment. In the example presented in the paper, the simulated human indeed chose 2 subtasks and left 8 to the robot (see line 635). We have run many variants of the misbalanced subtask assignment and generally observed “steeper robot learning

curves when more subtasks are assigned to the robot compared to the human and vice-versa more shallow learning curves when less subtasks are assigned to the robot” (see line 631). As for the illustration of this result in the manuscript we found the example of 2 subtasks chosen by the human to be most illustrative which is why we decided to show it in the manuscript. To avoid any misunderstanding, we added a statement to the caption of figure 4 “(6 subtasks with balanced assignment, 10 subtasks with balanced assignment, and 10 subtasks with 2 human and 8 robot assigned subtasks)”.
