# Supplementary material for: Human-robot collaborative task planning using anticipatory brain responses
Source: PLoS One. 2023 Jul 11;18(7):e0287958. doi: 10.1371/journal.pone.0287958 (PMC10335656; doi:10.1371/journal.pone.0287958)
Supplement: Attachment — Submitted filename: Response to Reviewers.pdf [file pone.0287958.s007.pdf]

Dear Editor and reviewers,

We thank the editor and reviewers for their constructive and insightful comments and recommendations which have helped further to significantly improve the manuscript. We have revised the manuscript (R3) according to the single left open comment from reviewer 3. Furthermore, we reviewed our reference list to ensure that it is complete and correct. Please note, that since the manuscript did not undergo any further changes, as the single reviewer 3 comment referred to Figure 1, the 'Revised Manuscript with Track Changes' is identical to the 'Manuscript'.

Reviewer 3:

All my comments have been addressed properly. However, Figure 1 is missing in the revised manuscript.

Response: The figure was indeed missing from the submission – we missed to notice that it was not properly processed by the EM pdf maker. Apologies for rendering the need for another round of revision. The figure is now accompanying the revision and addresses all points raised by the reviewer in the previous revision.
