# Supplementary material for: Human-robot collaborative task planning using anticipatory brain responses
Source: PLoS One. 2023 Jul 11;18(7):e0287958. doi: 10.1371/journal.pone.0287958 (PMC10335656; doi:10.1371/journal.pone.0287958)
Supplement: Attachment — Submitted filename: Review_PONE-D-21-32176_authors.docx [file pone.0287958.s004.docx]

**Review: PONE-D-21-32176**

**Neuro-cognitive measures of anticipated takeover situations enable human-robot collaborative task planning**

Dear authors,

Thank you very much for your submissions and for a clearly written paper. Overall, the paradigm proposed, and the technical approach has very positive approach. Nevertheless, there are three key aspects that need attention: the 1) neural signal explored, which involves a redefinition of the data segmented, 2) details about the methods and the protocol employed, and 3) the interpretation of the signal studied and the results. I do have a list of comments, I am sure most of them are easily solved by adding clarifications.

Finally, the authors present an analysis of the signals in the context of motor control at the end of the document that is on spot. This should further support the need for the authors to explore motor preparatory activity as a driver for their study.

**GENERAL COMMENTS**

1) Regarding the neural signal explored, it is not clear to me what the authors seek by analyzing ErrP activity in the context of anticipatory takeover. This is in part due to the absence of some key information regarding the experimental protocol (please see note about not finding any information regarding reference 60) but also due to how data is segmented prior to being analyzed (as defined in table 1). The use of ErrPs is intended for goal-oriented tasks, where errors are to be detected. Nevertheless, in the current paradigm, the authors do not define clearly errors guiding the subjects’ behavior. Moreover, most of the analysis in the paper (including the title) seem to focus on anticipatory behavior (as stated in the abstract: “prediction of the human anticipation”), but no anticipatory EEG activity (EEG measures of anticipatory behavior, mu rhythm, motor preparation, or anticipatory slow cortical potentials) is covered in the introduction, nor used to set the paradigm, extract data, analyze the results, discuss then and get to conclusions. This is a key element that need attention.

The authors are encouraged to explore the concept of movement preparation and EEG measures of anticipatory behavior, which seem to connect seamlessly with anticipatory takeover and the paradigm presented in this manuscript. Consider this: To truly analyze an anticipated takeover situation, it could be useful to focus not on a comparison between trial *t* and trial *t+1* but rather on the activity prior to the takeover event (prior to the robot onset due to a human button press). Motor anticipatory activity is expected to be present when a human will perform an action, and hence, this will be observed in the EEG activity some hundreds of milliseconds prior to the button press/robot movement onset.

There is a body of research in the field of EEG measures of anticipatory behavior and movement preparation (including the mu rhythm). If overall, the authors wish to focus on ErrPs, it is important to make clear for the reader, in the title, abstract and main body of the manuscript, why these are the signals explored and not preparatory/anticipatory rhythms. Please see the following citations for more info about movement preparation and anticipation:

- M. Rodrigo, L. Montesano and J. Minguez, "Classification of resting, anticipation and movement states in self-initiated arm movements for EEG brain computer interfaces," 2011 Annual International Conference of the IEEE Engineering in Medicine and Biology Society, 2011, pp. 6285-6288, doi: 10.1109/IEMBS.2011.6091551.

2) There are several portions of the experiment that need further details, so the experiment can be accurately reproduced by the readers; especially the takeover policy, a clear description of how the robot trajectories are defined (start, end, number of steps, etc), and how a trial is defined in the study.

3) The authors interpretation of the signal explored (ErrP vs. other possible signal) and the epoching/segmentation of the data leave gaps that need to be bridged. This affects all the paper sections, so it is recommended to revise them. It is especially important for the authors to explore the activity prior to the button push onset that represents a human takeover.

Please proofread the whole document, some words are repeated, and minor corrections are needed.

Finally, there seem to be some redundant references used throughout the document, such as over referencing prior work listing several publications of the authors for the same topic.

**SPECIFIC COMMENTS:**

**Abstract**

The authors mention in the abstract that this study allows “the prediction of the human anticipation of a robot-to-human takeover situation in single-trial.” but such outcome is yet to be completely accomplished. Specially since the classifier’s performance is nearly close to chance level. Moreover, it is important for the authors to clarify the real cognitive activity that is been study, which seems to be related to motor preparation, which indirectly reflects the future human takeover.

**Introduction**

It is recommended for the authors to include in the “Neuroengineering approaches to HRC” section studies that have covered EEG measures of anticipatory behavior and motor preparation since these seems to be the core of the signal explored by the authors.

The authors must include/explain how the ErrP signal represents elements related to an anticipated takeover. Is this because a takeover represents an error and hence the ErrP will label such discrepancy? Please clarify.

[Page 2, line 22] Please make clear if footnote 1 is taken from reference 7 or if it is the authors’. It is not clear based on how reference 7 is used in the manuscript (not in the footnote).

[Page 4, line 121] The authors mention the definition for ErrP from reference 41, dating 1997. It is recommended to incorporate the seminal work of Falkenstein et al., 1991 and Gehring et al., 1993 (reference 44 in the manuscript); who first identified and described the ErrP signal, later known as “response ErrPs”. Reference 41 in this manuscript, Miltner et al., 1997, described what are called “feedback ErrP”.

- Falkenstein, M. et al., 1991. Effects of crossmodal divided attention on late ERP components. II. Error processing in choice reaction tasks. Electroencep. Clin. Neurophysiol., 78, pp.447–455.
- Gehring, W.J. et al., 1993. A neural system for error detection and compensation. Psychol. Science, 4(6), pp.385–390.

**Experimental HRC study**

[Page 6, line 208] The authors seem to refer to the intermittent collaboration, so the figure referred seems to be Fig 1(b), not Fig 1(a). Please revise.

[Page 6. Line 216] The authors must include as part of the experimental paradigm an explanation of the criteria to select the positioning of the start and the end/target location, including the minimum number of steps required to reach the goal from the starting point (including metrics about the average and S.D. of the steps required), as well as how the spatial distribution of starting and ending points where balanced to avoid biasing the experiments. The authors mention this info is in reference 60 but such document is not currently available online (not Transactions on Biomedical Engineering, 2021). For reproducibility purposes, it is recommended to include this information in the manuscript (also please revise any typos in reference 60).

[Page 7. Line 237] For reproducibility purposes, it is recommended for the authors to include the positioning of the head (facing forward and parallel to the horizontal plane?) and their visual angles when following the robot’s possible trajectories.

[Page 7. line 243] If motor preparatory activity is not the focus on the study, the author must explain what conditions were taken into account to avoid EEG motor activity, and preparatory activity, that could affect the EEG recorded during the task.

[Page 7, line 249] For reproducibility purposes, the authors must clearly explain what the rules (beyond the key distribution in the iC scenario) for takeover were (takeover policy), both for the subjects and the robot. In the sequential collaboration, can the robot alternate directions or will always follow one direction and only change once reaching some established goals? In the intermittent collaboration, will the robot or human have a maximum number of possible takeovers? Can takeovers happen one after the other or each agent can and must at least control two consecutive steps? Please clarify in the manuscript.

[Page 8, line 290] It is recommended for the authors to clarify in section 2.3.4 “Timing validation“ and in the S1 Appendix (“Validation of robot motion timing” [Page 21, line 813]), if the validation experiment was performed per subject (specifying if done at the start or end of the recordings) or if it only took place once, with one subject, for the whole study across subjects. This clarification informs about the reliability of such validation.

[Page 8, line 293] The authors mention the concept of ‘anticipated’ but do not specifically explain what they mean until it is mentioned in the caption for Fig. 2. Please include in the ‘Experimental protocol’ a clear definition of what ‘anticipated’ means in the context of the experiment.

[Page 8, line 295] When the authors refer to “episodes”, it is recommended to properly explain what these represent. Are the episodes the possible directions the robot can take (4 in total) or do they represent a single trial (as later presented in table 1)? This is not fully clear. This information is necessary to replicate the methodology.

[Page 8, line 294] Please include in the manuscript information about the average time (and standard deviation) required to complete a trial, for each one of the scenarios. This information sheds light on the overlap of the epochs extracted, as well as on the total time experiment time (seems only available in page 16, line 580).

[Page 9, line 312, Section 2.5.1] It is necessary for the authors to include the length of the trials to fully understand its possible effects on the data epoching/segmentation. Under the consideration of a trial length too short, there is risk of high overlap between trial *t* and *t+1* using the 1200ms epoch length.

[Page 9, line 336] The authors must clearly explain the evidence supporting an analysis of the before and after epochs is the appropriate approach for exploring the human and robot takeover. It is not clear why the authors do not evaluate the activity prior to the human or robot takeover instead.

[Page 9, Table 1. and lines 313] In table 1 the authors us the variable (t) to represent the action before and after takeover or non-takeover, but later in section 2.5.1 (t) is used to represent the time in the epochs. This can be confusing for the reader. Please use different variables.

[Page 10, line 374] In section 2.5.4 it would be useful, for reproducibility purposes, to make clear to the reader what the classification outcomes are, including what the two classes represent, the y (x) values {-1, 1} as well as subscripts 1 and 2 in the equations (for the means and covariances).

[Page 10, line 390] The authors are encouraged to make clearer what the classes of the different classification problems are. It is mentioned later in the ‘Validation’ paragraph, but this information should be explicit in the ‘Binary classification’ section.

[Page 11, line 408, Validation] Could the authors explain what effect has in the decoding performance the use of the pick with replacement approach rather than balancing the conditions through pick without replacement (acknowledging this later approach is not appropriate since it would lead to folds with less trials than those needed to train a decoder using a features vector of 27 elements)?

**Results of experimental HRC study**

[Results] Please report the percentage of correct classification using false/true positives and false/true negatives, per subject, to better understand the effect of biased training data in the decoder performance. Moreover, please include in the results the overall classification chance per subject.

[Page 11, line 432, section 2.6.1] Are the analysis presented an aggregate of all available subjects or were they performed per subject? The authors must be clear about their approach and include in the manuscript if there were differences or similarities across subjects. This information is key for finding overall trends in the results presented.

[Page 12, below line 469, Figure 2 caption] The authors must make clear if the data presented belongs to one subject or if it is an aggregate of all subjects. Overall, it is expected that the results will be those of a representative subject.

[Page 12, below line 469, Figure 2 caption] The authors state in the caption that ‘anticipated’ in the experiment’s context refers to “the belief that a takeover situation will occur”. What is the evidence to make this statement?

Please consider the following: In the sequential collaboration scenario, takeover by the human only occurs once, in the transition boundary. But takeover seems to be defined by specific rules (the takeover policy that needs to be specified in the manuscript). For the belief of a takeover in this scenario, such belief does not really exists given the boundary itself and the current trajectory defined by the robot are clear elements that determine if a takeover should, and can, take place or not. In other words, a takeover is a function of the X-Y coordinates of the robot, and its past trajectory. Uncertainty of action only exists in the intermittent collaboration scenario. But it is again guided by the takeover policy provided to the subjects and robots. Without full access to the rules established it is impossible to understand how such expectation could be the driver of the activity studied in this experiment.

[Page 13, figure 3 caption] The authors are recommended to include in the figure 3 caption if the data presented belongs to before (*t*) or after *(t + 1)* an anticipated takeover situation.

[Page 13, line 474] The authors are encouraged to make clearer what the classes of the different classification problems are. If the two possible outcomes are HH or HR and RR or RH, please simplify the language and state this. Moreover, the style of presenting accuracy is confusing. The author present classification accuracy for one class and later for the other as if they were independent results. If these are related to true positive results for each possible class, please indicate it so but avoid using the ACCHH format since it misleads the reader to believe six different binary classifications took place.

[Page 13, Table 2] The authors are encouraged to change the format of the table, the names used for the column titles, and how the classification performance is presented. It is recommended to include the overall classification accuracy followed by the false and true positives, as well as false and true negatives. This allows better understanding of the classification and its tradeoffs. Please apply these recommendations to the S3 appendix information too.

[Page 13, line 482] To fully understand how close the simulation is to a real scenario, the authors are encouraged to clarify what scenario is simulated in this section, the sequential or intermittent collaboration. Please specify.

[Page 16, line 589] The authors used a theoretical value for the chance level (50%) while referring to using other empirical decoder parameters for their analysis. It is recommended to use the empirical chance level as well (54.3%) to truly “provide the closest estimate of how the robot reinforcement learning approach would perform in a real HRC experiment”. Upon making this change, it is recommended to update the figures affected by this value.

[Page 16, Figure 4 caption] For panel (c) the text reads “…dynamic task re-assignment after every 300 trials…” but the figure shows re-assignment after 40 episodes, that seem to represent 400 trials instead (based on text in a “trial = episode*10”). Please clarify and correct if necessary.

[Page 17, line 614] The authors state in the discussion section that “the neural responses observed and documented in the form of ERPs and ERSPs were surprisingly clean, e.g. comparable to screen-based stimuli which are easier to control” but it is not clearly defined if such signals represent grand averages of one specific subject or the overall average from 11 subjects, which radically modifies how clean the signals will look upon replication of the study. The authors are encouraged to clarify if the results are subject-specific and similar across subjects.

[Page 17, line 623] The authors state that “the ERP elicited … contains information about whether the human partner anticipated role changes of responsibility…” and base the before (t) and after (t+1) trial definition, and the subsequent results using this trial definition, to support their statement. Nevertheless, it is still not clear that the data analyzed indeed reflects an ERP that clearly contains anticipation. There is a wealth of research in EEG measures of anticipatory behavior and movement preparation that were not covered by the authors and that could clearly specify how to epoch the data to truly find ERPs and other signal characteristics to inform about the human partner intentions to takeover. The authors are encouraged to explore the EEG measures of anticipatory behavior and movement preparation literature.

**Discussion**

[Page 18, line 674] The authors state that “…in the present study, we investigate a predictive measure, e.g., a neuronal response informative of an anticipated situation, not the response to a situation that is currently happening. To the best of our knowledge, such kind of information retrieval from the human EEG for augmentation of HRC has rarely - if never - been proposed/investigated before.”

It is not clear the authors have provided enough evidence that the signal studied is a “neural response informative of an anticipated situation” nor have they shown that such signal is not motor preparatory activity related to the following button press action. In this context, there is strong evidence that a motor preparatory signal exists and has been explored in the context of human-robot collaboration. Below the authors can find a set of articles covering this area of study.

- Bozinovski and L. Bozinovska, "Anticipatory brain potentials in a Brain-Robot Interface paradigm," 2009 4th International IEEE/EMBS Conference on Neural Engineering, 2009, pp. 451-454, doi: 10.1109/NER.2009.5109330.
- R. Chavarriaga, X. Perrin, R. Siegwart and J. del R. Millán, "Anticipation- and error-related EEG signals during realistic human-machine interaction: A study on visual and tactile feedback," 2012 Annual International Conference of the IEEE Engineering in Medicine and Biology Society, 2012, pp. 6723-6726, doi: 10.1109/EMBC.2012.6347537.
- S. L. Norman, M. Dennison, E. Wolbrecht, S. C. Cramer, R. Srinivasan and D. J. Reinkensmeyer, "Movement Anticipation and EEG: Implications for BCI-Contingent Robot Therapy," in IEEE Transactions on Neural Systems and Rehabilitation Engineering, vol. 24, no. 8, pp. 911-919, Aug. 2016, doi: 10.1109/TNSRE.2016.2528167.
- Smyk NJ, Weiss SM and Marshall PJ (2018) Sensorimotor Oscillations During a Reciprocal Touch Paradigm With a Human or Robot Partner. Front. Psychol. 9:2280. doi: 10.3389/fpsyg.2018.02280
- Di Liberto, G.M., Barsotti, M., Vecchiato, G. et al. Robust anticipation of continuous steering actions from electroencephalographic data during simulated driving. Sci Rep 11, 23383 (2021). https://doi.org/10.1038/s41598-021-02750-w

[Page 19, line 728] The authors express that “…this neuronal measure provides information about the hidden states of a user, e.g., preferences and intentions. This novel information …”. It is important to clarify that the brain activity explored in this manuscript is likely the EEG desynchronization reflecting a motor preparation prior to a button push, which has been extensively study (see previous comment and the papers mentioned). For this reason, it is recommended for the authors to be careful using the concepts “preferences and intentions”, as well as “novel information”.

[Page 19, line 731] The comments expressed in the section “A neural correlate of anticipation of human-robot takeover situations?”, specially the first paragraphs, should be reassessed under the lens of the literature in motor anticipatory activity previously suggested.

**Conclusions**

The authors do explore neural correlates although its interpretation and the way the data is studied leaves gaps that need to be bridged.

[Page 20, line 790] The authors are invited to explore the use of motor preparation as a means for expressing an anticipatory takeover but paying careful attention to how the data is segmented and analyzed.

[Page 20, line 790] The authors are recommended to avoid using the statement “novel neuro-cognitive method” since the analysis of motor preparation, and their classification, in human-robot collaboration tasks has been previously explored.

**References**

[Page 25] Check the order of the authors in the reference section. As an example, in reference 35 the first author is DelPreto, not Hasani.

Some references, especially those from 2021, were not available online. Please double check the full references and add (in press) if those references are not yet published.

[Page 27] Reference 60 was not found in the IEEE Xplore site nor online. Double check the title, authors, and journal.

**Figures**

[Figure 2] The authors are encouraged to explore the EEG features of preparatory movement activity and mu-rhythms, which are very similar to what is presented in the bottom topoplot next to channel C4 in (b)

[Figures 2 and 3] The authors are encouraged to clarify if the results presented belong to a single subject (if so specifying its number) or are an aggregate of data from all subjects. Moreover, for readability purposes, the text in the figure should be sufficient to understand the image. Hence, the authors need to include in the C4, C3, FC1, and T7 plots the title (ERSPs) as well as axis labels and values (seems it is frequency and time as in ref. 69 but this must be confirmed by the authors and made clear to the readers). Moreover, it is not clear what the meaning of the curly brackets is. It seems to be pointing up to a specific time in the grand average ERP trace above it. It can be confused as zooming into the data at that specific time (200ms in (b), 650ms in (c), 850ms in (d)). It is fundamental to include color bars that indicate the meaning of the different colors in the topoplots and the ERSPs plots.

Finally, it is recommended for the authors to use the naming standard established in table 1. If ‘Robot takeover from human’ is represented by ‘HR’, then please include this ‘HR’ in the text on the left of the plot. The same applies for the ‘Human takeover from robot’ text at the bottom of the figure.

[Figure 3] Please follow for figure 3 the same recommendations about axis labels, color bars, title, the curly brackets and the use of the naming standard ‘RH’ that was mentioned for Figure 2.

[Figure 4] The author must clarify what an episode means and what it represents in the study, so it makes sense in the different Figure 4 plots. Moreover, the empirical values for ACC shown in (a) do not match the ones presented in table 2. Please clarify where these values come from and modify the plots as necessary using the correct values (e.g. based on Table 2, ACCHR is 51.4 for both iC or sC, respectively, not 56.1), including the empirical chance accuracy (54.7).

Extra figures: The authors are encouraged to present the topoplots, ERSPs and traces for the different subjects.
